# Supplementary material for: EMG-projected MEG High-Resolution Source Imaging of Human Motor Execution: Brain-Muscle Coupling above Movement Frequencies
Source: medRxiv. 2023 Jun 29:2023.06.23.23291825. Preprint. [Version 1] doi: 10.1101/2023.06.23.23291825 (PMC10327237; doi:10.1101/2023.06.23.23291825)
Supplement: Supplement 1 [file NIHPP2023.06.23.23291825v1-supplement-1.pdf]

medRxiv preprint doi: https://doi.org/10.1101/2023.06.23.23291825; this version posted June 29, 2023. The copyright holder for this preprint
(which was not certified by peer review) is the author/funder, who has granted medRxiv a license to display the preprint in perpetuity.
It is made available under a CC-BY 4.0 International license .

Supplementary Materials

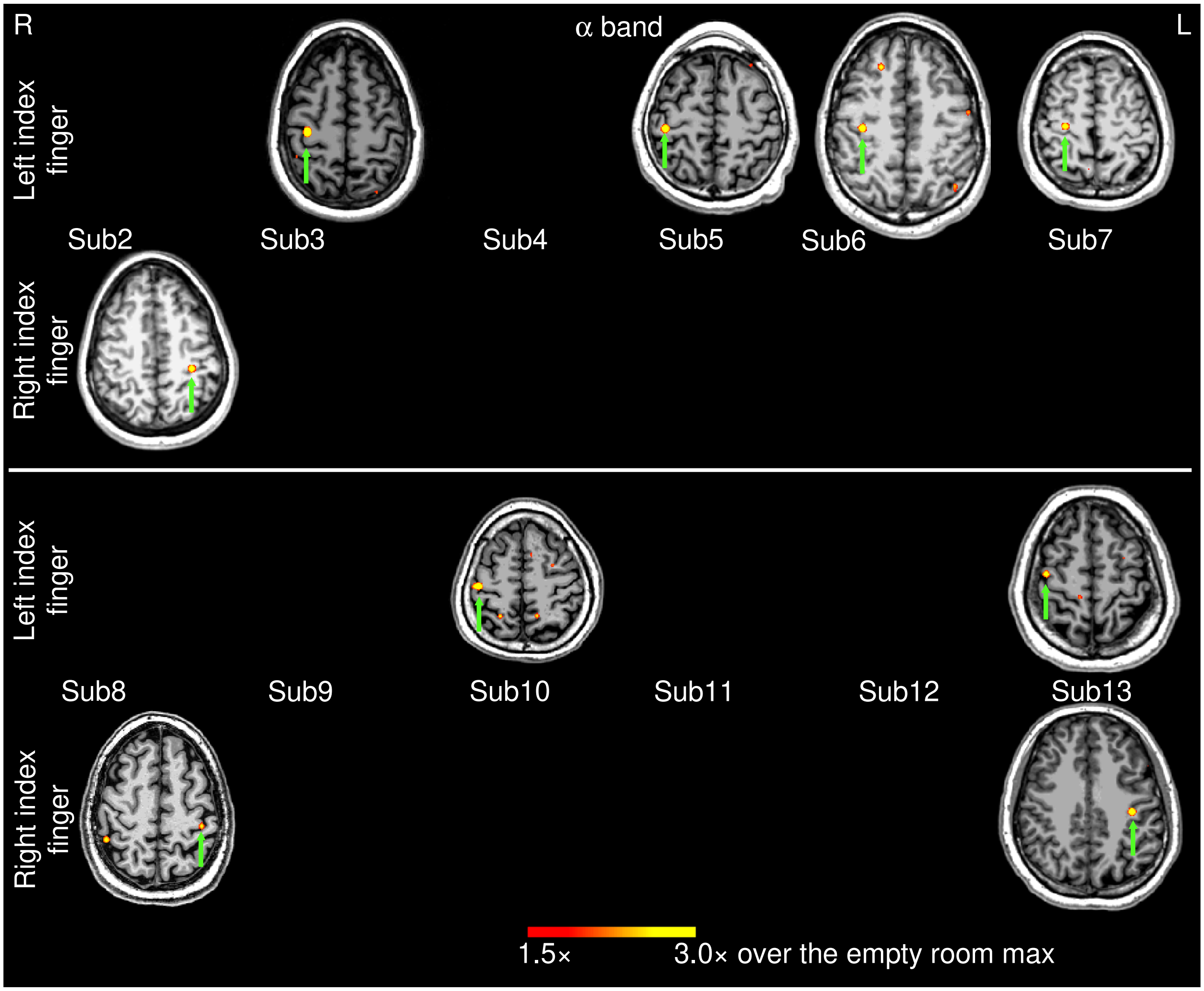

Figure S1: Movement-related alpha-band EMG-projected MEG source images for the remaining 12 healthy subjects. Significant primary motor cortex sources (green arrows) contralateral to left or right self-paced (~1 min) index finger movements. The color bar shows the activity threshold at 1.5× of the empty room maximum value, and saturation level at 3.0×. In 6 cases, contralateral primary motor source activity at or above the threshold was not observed. Sub = subject

medRxiv preprint doi: https://doi.org/10.1101/2023.06.23.23291825; this version posted June 29, 2023. The copyright holder for this preprint (which was not certified by peer review) is the author/funder, who has granted medRxiv a license to display the preprint in perpetuity.
It is made available under a CC-BY 4.0 International license .

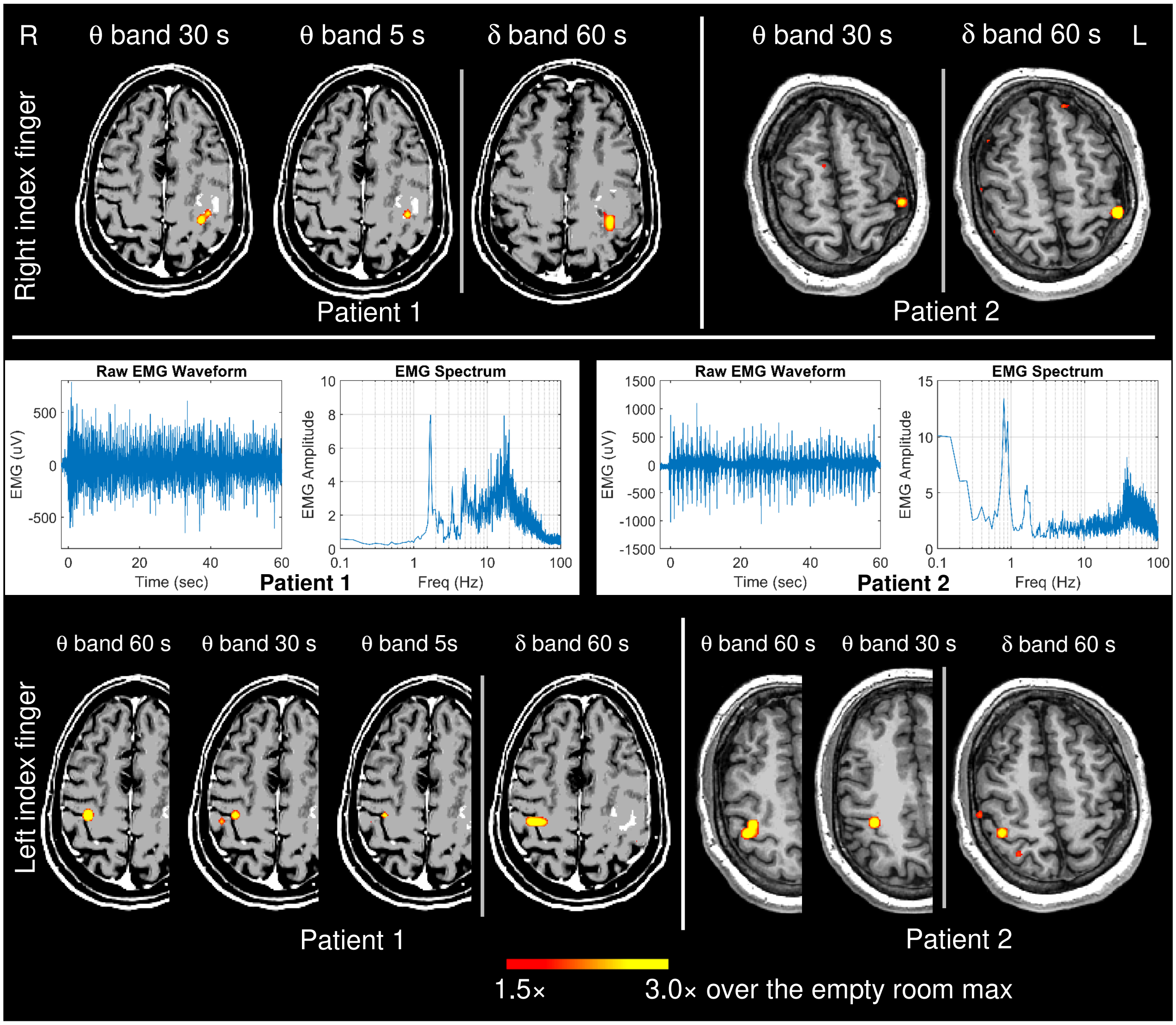

Figure S2: This figure provides supplementary results to Figure 5 in the main text. Top Panel: EMG-projected MEG source images of M1cortex in the affected left hemispheres due to right finger movement (affected hand) from the two clinical patients in theta and delta bands. In the theta band, M1 was localized in 30 sec and 5 sec time windows in Patient 1, but only the 30 sec time window in Patient 2. Delta-band M1 cortical activity (1 min EEG recordings) in the damaged hemisphere was localized in both patients. Middle Panel: the EMG waveforms and spectra from the left index finger movement (unaffected hand). Bottom Panel: EMG-projected MEG source images of M1 in the unaffected right hemispheres due to left finger movement from the patients in theta and delta bands. In Patient 1, theta-band M1 cortex activity was localized in the 60 sec, 30 sec and 5 sec time windows. In Patient 2, theta-band M1 activity was localized only in the 60 sec and 30 sec time windows. Delta-band M1 activity (1 min EEG recordings) was localized in the unaffected hemisphere in both patients.
